# Oropharyngeal dysphagia and amyloid beta pathology in the TgF344-AD rat model of Alzheimer’s disease

**DOI:** 10.3389/fnbeh.2026.1812480

**Published:** 2026-04-13

**Authors:** M. J. Cullins, A. K. Converse, L. M. Rowe, A. G. Hoerst, W. K. Hibbard, J. A. Russell, N. P. Connor, M. R. Ciucci

**Affiliations:** 1 Department of Otolaryngology-Head and Neck Surgery, University of Wisconsin-Madison, Madison, WI, United States; 2 University of Wisconsin-Madison Waisman Center, Madison, WI, United States; 3 Department of Communication Sciences and Disorders, University of Wisconsin-Madison, Madison, WI, United States

**Keywords:** Alzheimer’s, amyloid beta, dysphagia, swallowing, TgF344-AD

## Abstract

**Introduction:**

Dysphagia is a major consequence of Alzheimer’s disease (AD) that is understudied and undertreated. Neuropathology in AD occurs early in the disease progression, but little is known about pathologies underlying functional swallowing changes; this knowledge gap is a barrier to developing effective treatment. We hypothesized that an established AD rat model (TgF344-AD) would demonstrate significant deficits in oromotor/swallowing function versus Wild Type (WT) with corresponding amyloid beta pathology in brain structures critical to swallowing.

**Methods:**

Nine male TgF344-AD and 6 Wildtype Fisher 344 rats underwent deglutition assessments and PET imaging using the radiotracer [^11^C]PiB to assess brain and brainstem amyloid beta (Aβ) pathology at 11 months of age—a time point corresponding to early-middle stage AD progression. *A priori* brain regions of interest (ROIs) included those commonly associated with Aβ pathology and more specific swallowing associated structures such as brainstem nuclei and cortical motor areas. Deglutition was assessed using a videofluoroscopic swallow study and a pasta biting task.

**Results:**

Significantly increased levels of Aβ in the AD group were found in regions critical to swallowing motor control including the secondary motor area, thalamus, nucleus ambiguus, and hypoglossal nuclei. The AD group demonstrated significant changes in aerodigestive coordination, including delayed swallow onset, increased apnea duration, and increased frequency of aberrant post-swallow inhale pattern that was correlated with nucleus ambiguus Aβ levels. The AD group also exhibited altered oral processing including reduced bolus size and mastication rate.

**Conclusion:**

The TgF344-AD rat model of Alzheimer’s exhibits robust changes in oral processing and respiratory-swallow coordination that parallel clinical AD dysphagia. At this early-middle stage timepoint, Aβ pathology is primarily impacting cerebral swallowing networks as well as the nucleus ambiguus and hypoglossal nuclei in the brainstem. Our finding of increased Aβ in the nucleus ambiguus warrants further study as this motor nucleus plays a role in swallowing, respiration, and vocalization—all factors that are known to be impacted by AD in the clinical population.

## Introduction

Dysphagia is a frequent comorbidity of Alzheimer’s disease (AD) (Simões et al., 2020), associated with reduced quality of life, malnutrition, and death from pneumonia (Mira et al., 2022; Chouinard et al., 1998). Swallowing impairments can appear early in the disease and are progressive, with dysphagia signs and symptoms becoming more pronounced in later stages of the disease (Simões et al., 2020; Parlak et al., 2022). The progression of dysphagia may contribute to an overall decline in health, with malnutrition exacerbating both cognitive and physical decline (Parlak et al., 2022). Thus, early dysphagia intervention has the potential to delay quality of life decline and corresponding increased health care needs and costs (Tian et al., 2013). However, our ability to develop effective interventions for AD dysphagia is limited by a poor understanding of the underlying neuropathology of swallowing dysfunction, especially in the earlier stages of the disease (Andrade-Guerrero et al., 2024; Humbert et al., 2010).

The neural control of swallowing is distributed throughout the CNS and includes both cortical and subcortical networks (Mistry and Hamdy, 2008; Cheng et al., 2022). Reduced activation of the cortical swallowing network, revealed by BOLD fMRI, precedes clinical dysphagia diagnosis, yet is associated with subclinical biomechanical changes (Humbert et al., 2010). Swallow pattern generating networks and motor nuclei are located in the brainstem; known AD neuropathology in the brainstem includes early volume loss (Lee et al., 2015; Jacobs et al., 2021) and neurofibrillary tangles (Ehrenberg et al., 2017; Parvizi et al., 2001), yet the role of AD brainstem changes on swallowing function has not been established.

The TgF344-AD rat model of Alzheimer’s disease exhibits a spectrum of clinically relevant AD neuropathologies such as amyloidosis, tau pathology, and neuronal loss as well as corresponding behavioral changes that progress with age (Cohen et al., 2013; Saré et al., 2020). This model allows for the preclinical evaluation of the neurophysiological underpinnings of AD dysphagia at all stages of disease progression.

A previous pilot study established clinically relevant early changes in swallowing and oral processing in the TgF344-AD model at 11 months of age (Rowe et al., 2024). Understanding the neuropathologies driving these changes in swallowing function may identify potential biological targets for early interventions seeking to delay or prevent decline in swallowing function and associated maladies. This timepoint corresponds to an early-middle stage of AD progression in the TgF344-AD model; rats exhibit a range of behavioral, neurochemical, and neuropathological changes including moderate levels of hallmark AD pathology amyloid beta (Aβ) (Cohen et al., 2013; Saré et al., 2020).

Aβ pathology has been identified as one potential factor in motor decline in AD (Andrade-Guerrero et al., 2024), thus we sought to determine whether early changes in mastication and swallowing are associated with Aβ pathology in brain regions that have a role in the control of swallowing.

In this exploratory study we used PET imaging to quantify Aβ, a standard clinical approach. We hypothesized that 11-month-old TgF344-AD rats would demonstrate significant changes in oromotor and swallowing function relative to Wild Type (WT) with corresponding Aβ pathology in brain structures critical to swallowing.

## Methods

### Animals

All experiments were approved by the University of Wisconsin School of Medicine and Public Health Animal Care and Use Committee (IACUC) and conducted in accordance with the National Institutes of Health Guide for the Care and Use of Laboratory Animals (National Research Council, 2011). ARRIVE 2.0 guidelines have been incorporated into the design and reporting of this study (Percie du Sert et al., 2020).

Nine male TgF344-AD and 6 male Wildtype Fisher 344 rats underwent deglutition assessments and PET imaging to assess brain and brainstem Aβ pathology at 11 months of age—a time point in this model that corresponds clinically to early middle-stage AD. Rats were either sourced from Rat Resource & Research Center (RRRC, University of Missouri, Columbia, MO) or generated from breeding pairs of male TgF344-AD (RRRC) and female WT F344 (Envigo). Specifically, 6 WT and 4 TgF344-AD rats came from RRRC and 5 TgF344-AD rats from the breeding core; control rats were not littermate controls. Rats were housed in standard polycarbonate cages with corncob bedding in pairs until 10 months of age when they were moved to single housing with enrichment due to aggression in the TgF344-AD rats at this age. From arrival rats were maintained on a reversed 12 h light/dark cycle to ensure experiments were conducted during their time of normal activity. Overall health and weight were carefully monitored and no gross motor or dental issues were noted.

All testing and imaging were completed over the course of a week. Deglutition was assessed using a videofluoroscopic swallow study (VFSS) and a vermicelli pasta biting task. Rats underwent acclimation to the behavioral testing procedures and food sources and were food restricted prior to testing. At all other times rats were provided with *ad libitum* food (Standard rat chow; 8604 Rodent Diet, Inotiv) and water. Each procedure was performed on a separate day and in the following order: pasta biting, PET scan, then VFSS, with the exception of two cases in which PET scans were delayed until after VFSS due to scanner availability.

### PET imaging

High resolution PET imaging was used to quantify early changes in Aβ throughout the brain and brainstem using the radiotracer [^11^C]PiB; uptake was quantified as SUVr (standardized uptake value ratio), normalized to cerebellum. *A priori* brain regions of interest (ROIs) included regions commonly associated with amyloid beta pathology and more specific swallowing associated structures such as brainstem nuclei and cortical motor areas.

PET neuroimaging was performed as previously described (Converse et al., 2024, 2025). The amyloid-beta plaque ligand [^11^C]PiB was synthesized as described previously (Johnson et al., 2014). Under isoflurane anesthesia, rats were scanned with a microPET P4 tomograph (Siemens, Knoxville, TN) with 2 mm FWHM resolution (Tai et al., 2001). Rats were placed parallel to the axis of the scanner bore, with their heads fixed by tooth bars as well as ear bars, which were positioned at the center of the axial field of view. After a ^57^Co transmission scan, radiotracer was injected intravenously with a target dose of 37 kBq/g (molar activity 878 ± 331 MBq/nmol, mean ± SD, *n* = 15), and emission data were collected for 90 min. Images were reconstructed in 1-min frames to yield measures of radioactivity concentration (Bq/mL).

**Figure 1:**
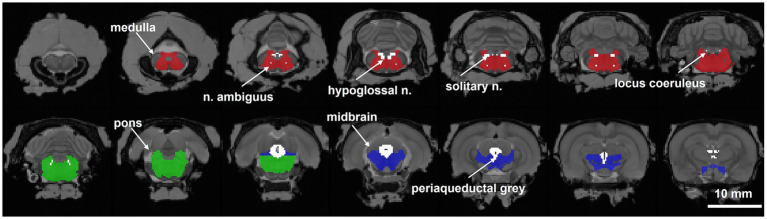
Brainstem regions for analysis of PET images. Eight *a priori* brainstem regions of interest (ROIs) were identified and delineated in the Waxholm space atlas (Papp et al., 2014). The periaqueductal gray is defined in the Waxholm space atlas. The medulla (red), pons (green), and midbrain (blue) divisions of the Waxholm brainstem, nucleus ambiguus, hypoglossal nucleus, solitary nucleus, and locus coeruleus were all drawn with reference to Paxinos and Watson (1998).

Summed images acquired 0–10 min post-injection of tracer were aligned by nine degrees of freedom to the Waxholm rat brain atlas (Papp et al., 2014), and the same transformations were applied to the 90 min 4D images. In addition to the ROIs provided with the Waxholm atlas (V1.01:neocortex, hippocampus, periaqueductal gray, thalamus, perirhinal cortex; V4: primary motor cortex, secondary motor cortex, amygdaloid area), several ROIs were delineated in-house, namely, medulla, pons, midbrain, nucleus ambiguus, hypoglossal nucleus, solitary nucleus, and locus coeruleus (Figure 1). Time-activity curves relative to injected dose per body weight (standardized uptake value, SUV) were calculated for each ROI. For each ROI, 50–70 min average SUVs were scaled to the cerebellum SUV yielding relative standardized uptake values (SUVr) that were used in the statistical analysis.

### Videofluoroscopic swallow study (VFSS)

**Figure 2:**
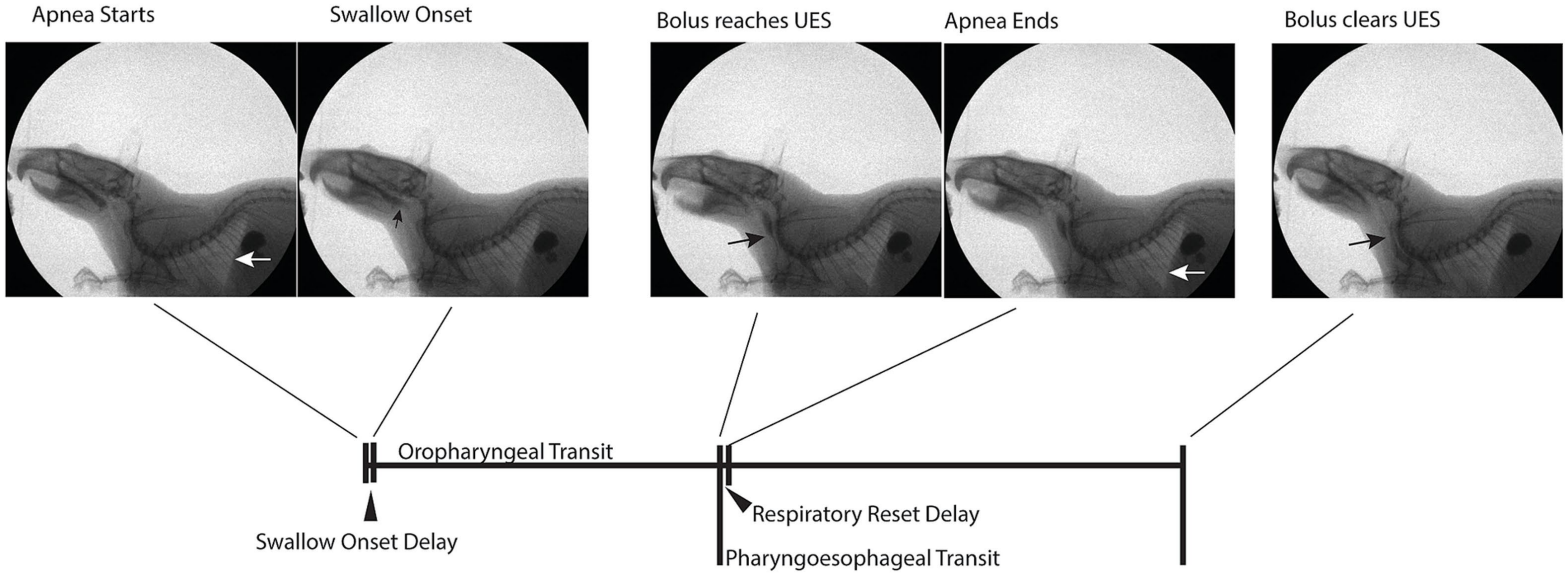
Videofluorographic swallowing study (VFSS) illustration of event timing underlying outcome measures. Apnea start and end were determined by diaphragm movement (white arrow). Black arrows indicate points of interest for bolus progression over the course of the swallow.

All rats underwent an established videofluoroscopic swallow testing protocol (Russell et al., 2013; Cullins and Connor, 2019; Kletzien et al., 2019), using a well-tolerated mixture of 5 g peanut butter and 5 mL barium sulfate (EZ-M Varibar Nectar), following an overnight food restriction to increase motivation to participate in the task. Videofluoroscopic data were collected at 60 frames per second, with a maximum of 5 min feeding time. Bolus area and diaphragm movements were quantified in ImageJ to derive functional outcome measures of kinematics and aerodigestive function (Figure 2 and Table 1). Bolus area (mm^2^) was measured at the frame of the bolus first touching the upper esophageal sphincter (UES). Diaphragm movement, an indicator of respiration, was used to determine respiratory direction (inhalation/expiration) and apnea duration and timing (Rowe et al., 2021). Investigators blinded to experimental group conducted off-line analysis of all visible swallows (minimum of 3 per animal, average of 12 swallows per animal with a standard deviation of 5) within feeding period. The average number of swallows analyzed per animal was not significantly different between groups (AD = 13.7 ± 5.8, WT = 10.0 ± 2.9, *p* = 0.19, 2-tailed student’s *t*-test). The resolution of time-based measures was limited by frame rate, with each frame representing 16.7 ms. Average values for each rat and group were calculated in milliseconds.

**Table 1:** VFSS outcome definitions.

VFSS outcome	Definition
Residue	0 = No residue pre or post swallow 1 = residue pre-swallow that is cleared post-swallow 2 = residue post-swallow only 3 = residue pre- and post-swallow
Post-swallow inhales	Percentage of swallows in which aberrant post-swallow inhale is observed (diaphragm pulls down caudally/pleural space expands after obligatory swallow apnea)
Oropharyngeal transit duration	Duration from swallow onset (bolus head enters posterior oropharynx and contacts posterior pharyngeal wall) until bolus head touches UES
Pharyngoesophageal transit duration	Duration from bolus head first touching UES, until bolus passes completely through UES to enter proximal esophagus
Inter-swallow interval	Duration between swallow onsets
Apnea duration	Duration where diaphragm is not moving toward inhale or exhale position for at least 2 consecutive video frames
Swallow onset delay	Duration between onset of apnea (cessation of diaphragm movement) and onset of the swallow (bolus head entering posterior oropharynx)
Respiratory reset delay	Duration between offset of the OP swallow (bolus head first touching UES) versus offset of apnea (first frame of diaphragm movement toward post-swallow respiration).

Outcome measures defined in Table 1 were derived from clinical reports of AD dysphagia, adapted for use in rats, and selected based on a pilot study of VFSS in the TgF344-AD model. Clinically, early dysphagia characteristics in AD include a prolonged oral stage with inefficient mastication, delayed swallowing reflex, residue, and prolonged apnea (Mira et al., 2022; Parlak et al., 2022; Seçil et al., 2016). Therefore, our expected outcomes included slower oral processing (mastication rate, swallow onset delay, oropharyngeal transit duration) (Mira et al., 2022), increased residue, and smaller bolus area.

### Pasta biting

Mastication testing was performed using a vermicelli pasta-biting task commonly used in the rat to assess motor deficits (Krekeler and Connor, 2017; Allred et al., 2008; Plowman et al., 2013; Kane et al., 2011; Tennant et al., 2010). Rats were provided with uncooked vermicelli pasta and audio recordings were used to quantify the mastication rate (Avisoft SASLab Pro). The average mastication event rate, including bites and chews, was determined across 5 pasta piece trials (7 cm pieces).

### Statistical analysis

Statistical analyses were performed using SPSS (IBM v31). Between group comparisons of neuroimaging and behavioral data were performed using a Mann–Whitney *U* test (exact 2-sided test, *α* = 0.05). We did not use a correction for multiple testing due to the exploratory goal of this study.

VFSS timing elements, including oropharyngeal transit, pharyngoesophageal transit, swallow onset delay, and apnea duration, were further evaluated by ANCOVA to account for the effects of bolus size by including it as a covariate.

Correlational analysis between PiB levels of *a priori* ROIs and functional deglutitive behaviors (swallowing and mastication) were assessed using Spearman’s rho due to small sample sizes and unequal variances (2-tailed, *α* = 0.05). Comparisons were limited to variables with significant group level differences. Outliers more than 1.5 times the interquartile range outside the upper or lower quartile (Brant, 1990; Tukey, 1977) were excluded from correlation analysis (4 total outliers; 1 each from bolus area, post-swallow inhale, swallow onset delay, and apnea duration). Data, linear fit model, and confidence intervals were plotted in Mathematica (Wolfram 14.2).

## Results

### PET amyloid beta imaging: *a priori* ROI analysis

**Figure 3:**
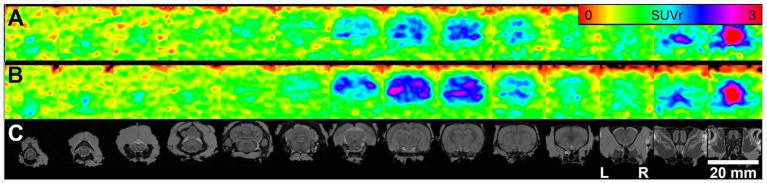
[^11^C]PiB PET imaging of amyloid beta in rat brain. Average PET images of **(A)** WT rats (*n* = 6) and **(B)** TgF344-AD rats (*n* = 9). PET images show radioactivity scaled to cerebellum (SUVr), 50–70 min post-injection of radiotracer. **(C)** PET images were fit to the Waxholm Space MRI template; 2.5 mm coronal slices are shown from posterior (left) to anterior (right), aligned with corresponding PET data in A and B.

The presence of amyloid beta throughout the brain was quantified by imaging the uptake of the amyloid beta ligand [^11^C]PiB (Figure 3). Relative standardized uptake values (SUVr) of PiB were evaluated in 16 *a priori* regions of interest, including both swallowing specific regions and brain regions known to exhibit Aβ plaques. Aβ levels, as indicated by [^11^C]PiB uptake, were significantly higher in AD than WT across the whole brain (AD = 1.49 ± 0.07, WT = 1.31 ± 0.05 SUVr, mean ± SD, *p* = 0.002), and in regions well-established for early Aβ pathology in AD including the hippocampus (AD = 2.09 ± 0.15, WT = 1.62 ± 0.06 SUVr, *p* < 0.001), perirhinal cortex (AD = 2.04 ± 0.13, WT = 1.44 ± 0.13 SUVr, *p* < 0.001), and neocortex (AD = 1.46 ± 0.11, WT = 1.21 ± 0.05 SUVr, *p* < 0.001).

**Figure 4:**
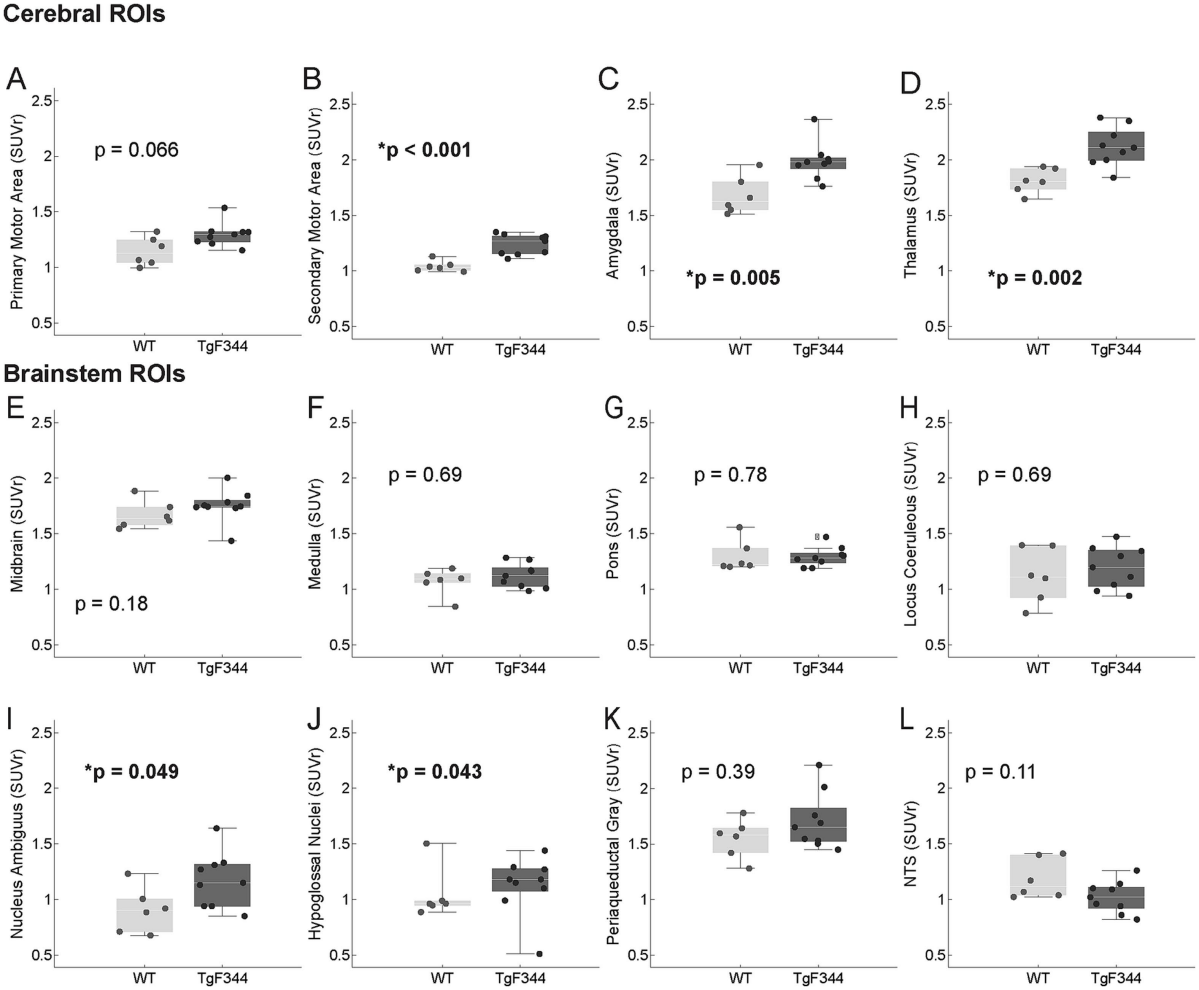
AB PET swallowing related ROIs. PET imaging indicated significantly higher AB in the AD group compared to WT in all forebrain regions except the primary motor area **(A–D)**. Brainstem ROIs **(E–L)** were not significantly different between the AD and WT groups, with the exception of the nucleus ambiguus **(I)** and hypoglossal nucleus **(J)**, in which PET imaging indicated significantly higher AB in the AD group.

We also found significantly higher Aβ levels with AD in swallowing related cerebral regions: secondary motor area, thalamus and amygdala (Figure 4). Of the 8 brainstem regions evaluated, only the nucleus ambiguus and hyopoglossal nucleus showed increased PiB uptake in the AD vs. WT group (ambiguus: AD = 1.17 ± 0.25, WT = 0.90 ± 0.20 SUVr, *p* = 0.0496; hypoglossal: AD = 1.20 ± 0.14, WT = 1.04 ± 0.23 SUVr, *p* = 0.043).

### VFSS results

**Figure 5:**
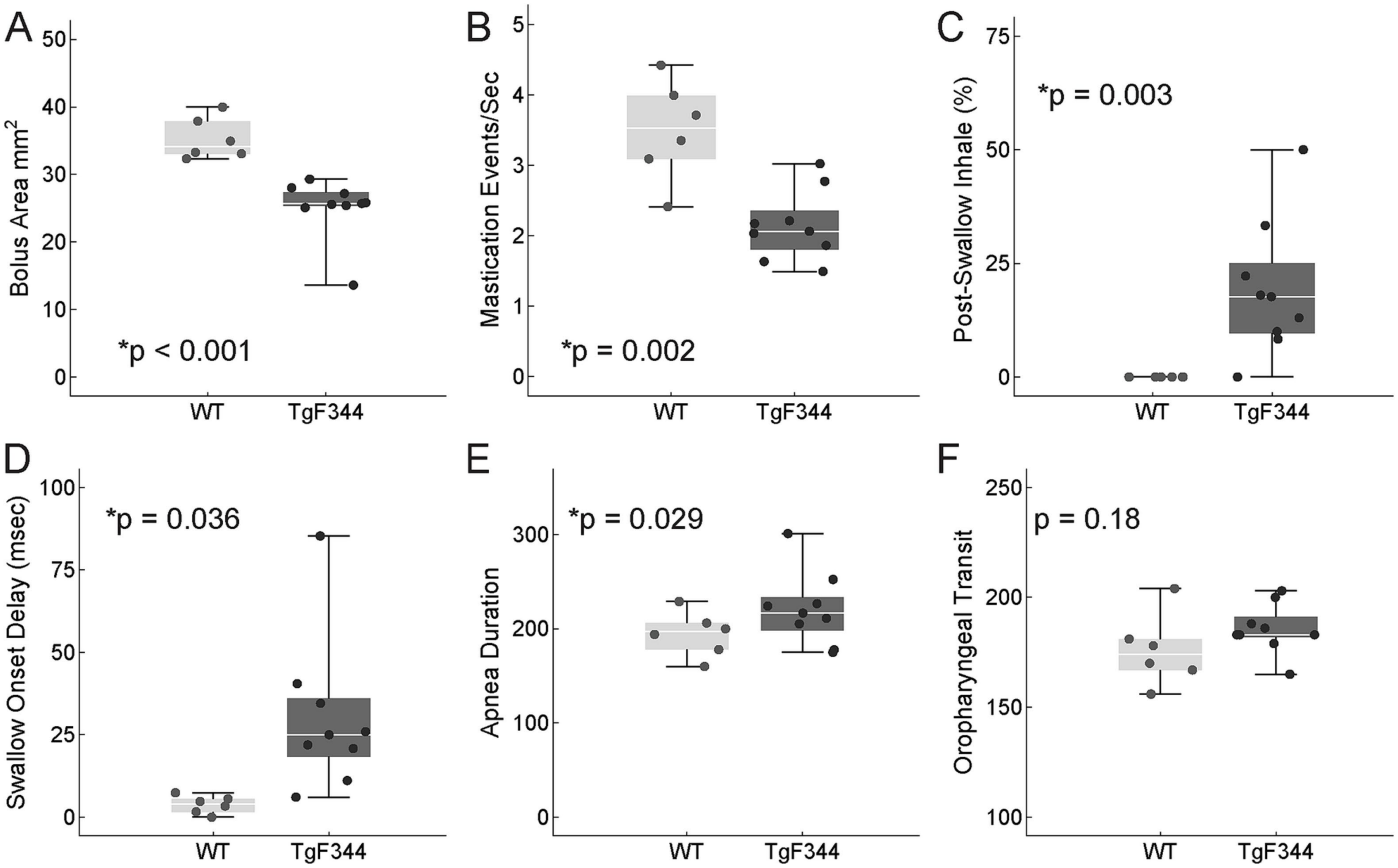
Functional deglutition assessments. Swallowing and mastication function was assessed by VFSS and a pasta biting task, respectively. The AD model was associated with a significantly **(A)** smaller bolus area, **(B)** slower mastication rate, **(C)** higher percentage of post swallow inhales, **(D)** larger swallow onset delay, and **(E)** longer apnea duration during swallowing. Oropharyngeal transit duration was not significantly different between groups **(F)**.

Nine VFSS measures were assessed; these factors were selected based on clinical swallowing changes reported in the literature of early-stage AD dysphagia and a pilot study of swallowing changes in the TgF344-AD model (Tables 1, 2). VFSS timing elements, including oropharyngeal transit, pharyngoesophageal transit, swallow onset delay, respiratory reset delay, and apnea duration were evaluated with bolus size as a covariate. Apnea duration and the swallow onset delay (time between the start of apnea and the initiation of swallowing) were significantly longer in the AD group than WT (Figure 5 and Table 2). Post-swallow inhalations, an aberrant respiratory-swallow coordination pattern, was exclusively found in the AD group (AD = 19 ± 15%, WT = 0 ± 0%, M ± SD, *p* = 0.003). The AD group also swallowed significantly smaller boluses (AD = 25.1 ± 4.5, WT = 35.2 ± 3.0, *p* < 0.001). No significant differences were found for residue score, inter-swallow interval, oropharyngeal transit duration, pharyngoesophageal transit duration, or respiratory reset delay (Table 2).

**Table 2:** Behavioral data.

Behavioral measure	WT	AD	*p*-value
Bolus area (mm^2^)	35.2 ± 3.0	25.1 ± 4.5	*p* < 0.001*
Residue score	0.90 ± 0.83	0.62 ± 0.75	*p* = 0.69
Post-swallow inhalation (%)	0 ± 0	19 ± 15	*p* = 0.003*
Mastication rate (events/s)	3.49 ± 0.71	2.14 ± 0.49	*p* = 0.002*
Inter-swallow interval (sec)	3.5 ± 1.0	3.7 ± 1.0	*p* = 0.77
Oropharyngeal transit duration (ms)^†^	170 ± 10	192.6 ± 7.6	*p* = 0.18
Pharyngoesophageal transit duration (ms)^†^	414 ± 66	311 ± 52	*p* = 0.36
Apnea duration (ms)^†^	161 ± 55	251 ± 17	*p* = 0.029*
Swallow onset delay (ms)^†^	2.0 ± 6.0	27.0 ± 5.2	*p* = 0.036*
Respiratory reset delay (ms)^†^	21 ± 9	25 ± 8	*p* = 0.29

Significant *p*-values indicated by asterisk and bold text.

^†^Indicates measures evaluated with bolus size as a covariate (bolus area = 30.3 mm^2^), estimated marginal mean ± SE; all other measures compared by Mann–Whitney *U* test and report the data mean ± SD. UES, upper esophageal sphincter.

### Pasta biting

We found a significantly slower pasta mastication rate in the AD group (AD = 2.14 ± 0.71, WT = 3.50 ± 0.95 events/s, mean ± SD, *p* = 0.002, Figure 5B).

### Correlations

**Figure 6:**
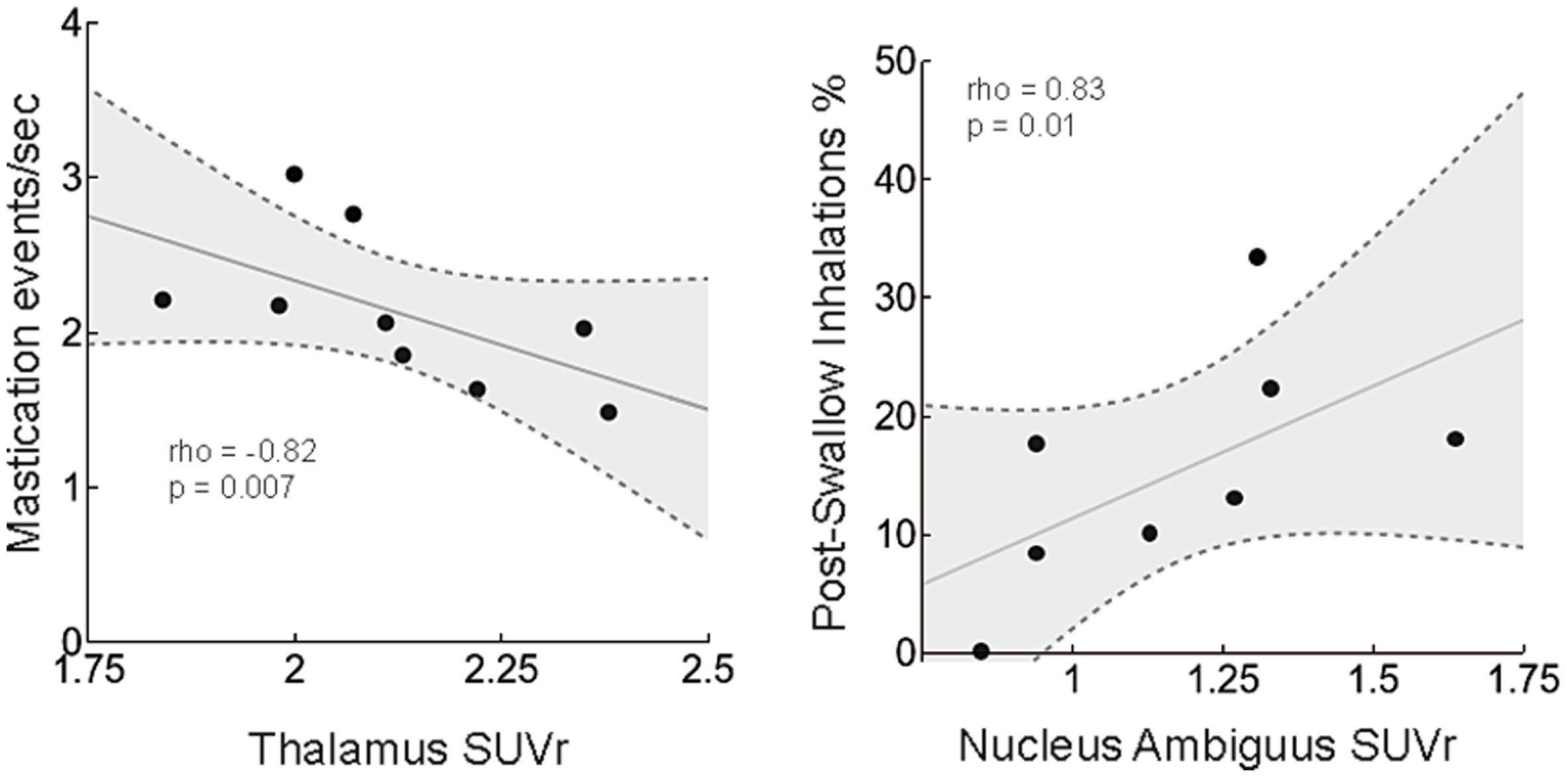
Correlations between amyloid beta and deglutitive function. Increased amyloid beta (PIB uptake) in the thalamus was correlated with decreased mastication rate. In the nucleus ambiguus, increased amyloid beta was associated with an increased percentage of post-swallow inhalations. Bivariate correlation analysis was carried out for all ROIs and functional behaviors that were significantly different between the AD and WT groups. Dashed lines with gray fill indicate 95% confidence intervals.

Strong significant correlations within the AD group between deglutitive behaviors and PiB uptake in *a priori* ROIs were found (Figure 6). The pasta mastication rate was negatively correlated with PiB uptake in the thalamus (rho = −0.82, *p* = 0.007), such that higher Aβ levels were associated with slower mastication. Post inhalation swallow percentages were positively correlated with the nucleus ambiguus PiB uptake (rho = 0.83, *p* = 0.01), indicating higher Aβ levels in the nucleus ambiguus were associated with more frequent post-swallow inhalations.

### PET amyloid beta imaging: extended exploratory analysis

Our initial analysis focused on *a priori* analyses to limit false discoveries, but PET scans generated data on PiB uptake throughout the brain allowing for a more extensive exploratory analysis of the model. Using brain regions delineated by both versions 1.01 and 4 of the Waxholm space atlas, we generated a data set of PiB uptake values, compared the WT and AD groups by *t*-test, and ranked regions by *p*-value (Supplementary data). Regions with highly significant Aβ levels include the perirhinal cortex, hippocampus, auditory regions, olfactory/taste regions (piriform cortex) and thalamic/motor regions. Taste regions may be of important consideration for swallowing changes.

## Discussion

This is the first study to use PET imaging of the radiotracer [^11^C]PiB to detect Aβ in the brain and brainstem in an early stage TgF344-AD model. We established changes in ingestive behaviors associated with Aβ pathology in brain structures critical to swallowing. At this early-middle stage timepoint, Aβ pathology (detected by PET imaging) is primarily impacting cerebral swallowing networks along with the nucleus ambiguus and hypoglossal nuclei in the brainstem.

### Functional deglutition changes

AD dysphagia signs and symptoms are progressive, with the number and severity of signs and symptoms increasing later in the disease progression (Mira et al., 2022; Seçil et al., 2016; Yildiz et al., 2015). Consistent with clinical findings in early AD, we found functional changes in measures related to the oral stage of swallowing (Priefer and Robbins, 1997) and respiratory-swallow coordination (Seçil et al., 2016).

Significant differences related to the oral stage of swallowing included a slower mastication rate and smaller bolus area. Slower mastication rate is consistent with clinical findings of prolonged oral phase durations (Priefer and Robbins, 1997) and impaired masticatory performance in patients with AD (Seçil et al., 2016). Slower chewing and consuming less material per swallow suggest a greater level of effort and longer meal time required to take in nutrition. These factors may contribute to malnutrition as the disease progresses. All rats regardless of genotype eat the barium peanut butter mixture enthusiastically, so we consider it unlikely that palatability of the mixture influenced these findings.

Impaired respiratory-swallow coordination, which may increase the risk of aspiration and pneumonia, has been reported in clinical populations with AD and mild cognitive impairment (MCI) (Seçil et al., 2016; Oku et al., 2021). Specifically AD and MCI are associated with greater apnea duration, swallow latency (Seçil et al., 2016), and swallowing during inspiration (Oku et al., 2021)—all of which were replicated in our study with similar significant measures (apnea duration, swallow onset delay, and post swallow inhale % respectively Figure 5).

In contrast to our findings, a previous study in the TgF344-AD model found no changes in a variety of respiration measures at a broad timepoint of 8–11 months of age (Lucking et al., 2019). Our findings may differ because respiratory swallow coordination changes may precede more general respiratory changes, or signs may be emerging only at the later 11 month time point.

Further evidence of early changes in TgF344-AD aerodigestive tract function has been reported, with vocalization changes occurring as early as 9 months (Rudisch et al., 2024).

### Amyloid PET

Aβ pathology has been identified as one potential factor in motor decline in AD (Andrade-Guerrero et al., 2024), thus we sought to determine whether early changes in oromotor function and swallowing are associated with Aβ pathology in brain regions that play a role in the control of swallowing.

### Cerebrum

Increased Aβ levels ([^11^C]PiB uptake) in areas such as the cortex and hippocampus are in agreement with previous histological and imaging studies (Cohen et al., 2013; Fowler et al., 2022; Burgos-Puentes et al., 2026). Altered activation of swallowing related cortical areas has been reported in early AD in a clinical study that used BOLD MRI imaging (Humbert et al., 2010). We found increased Aβ levels in several swallowing related cerebral ROIs including the secondary motor area, thalamus, and amygdala. Aβ levels in the thalamus were correlated with average mastication rate, such that higher Aβ levels were associated with slower mastication (Figure 6A). The thalamus relays orofacial sensorimotor information, including jaw proprioceptive signals (Yoshida et al., 2017; Isogai et al., 2012), and was among the regions identified as significant for both fMRI BOLD signal (Onozuka et al., 2002) and function connectivity (fcMRI) during rhythmic chewing in humans (Quintero et al., 2013). We took a conservative approach to correlation analysis, yet it is important to interpret correlations of this study cautiously due to the small sample size, number of comparisons, and intercorrelations among brain regions for Aβ PET values.

A previous study (Saré et al., 2020) in the TgF344-AD model found particularly high Aβ concentrations in the hippocampus and perirhinal cortex, which are involved in memory learning and spatial navigation, and the piriform cortex, a region that processes olfactory and taste information. Our PET imaging results were consistent with these findings and additional navigation and olfaction associated brain regions with elevated Aβ (PiB uptake) were found (Supplementary data). Additionally, we identified elevated Aβ levels in brain regions associated with auditory function including the primary and secondary auditory areas; central auditory processing disorder has been identified as an early onset impairment in people with AD (Gates et al., 2008; Tarawneh et al., 2022).

The piriform cortex, a region that processes olfactory and taste information, was among the brain regions with the highest PiB uptake in the AD as compared to the WT group (Supplementary data). Reduced taste has been clinically associated with AD (Kouzuki et al., 2020) and may reduce interest in food, contributing to malnutrition. Additionally, loss of taste may directly impact swallowing function as taste has been shown to influence swallowing biomechanics (Dietsch et al., 2019; Abdul Wahab et al., 2011). In future work, quantification of taste changes and their relationship to ingestive behaviors may be possible in the TgF344-AD model.

There is no decisive list of brain regions that are critical to swallowing; swallowing is a complicated sequence of muscle activations relying on integration of sensory feedback, and a combination of volitional and pattern generator driven motor activity arising from a complex network distributed throughout the brain and brainstem. In our extended analysis of Aβ levels throughout the brain (Supplementary data), additional brain regions that were highly ranked for significant Aβ and have been associated with swallowing function may be of interest for future research, such as the primary and secondary somatosensory areas, globus pallidus, granular insular cortex, entopeduncular nucleus, and caudate putamen (Cheng et al., 2022; Dehaghani et al., 2016; Kim et al., 2025; Malandraki et al., 2009; Jang et al., 2017).

### Brainstem

We found significantly higher Aβ levels in the nucleus ambiguus and hypoglossal nucleus of the AD group. The nucleus ambiguus is a pair of motor nuclei in the medulla oblongata that contribute to swallowing, speech, and respiration (Petko and Tadi, 2025; Yajima and Hayashi, 1999)—all factors that are known to be impacted by AD in the clinical population. Additionally, the nucleus ambiguus is among the brainstem nuclei identified as selectively vulnerable to AD—positive for both Aβ and phosphorylated tau immunostaining in post-mortem AD brains (Parvizi et al., 2001). In the current study, the Aβ levels in the nucleus ambiguus were significantly correlated with a swallowing respiratory pattern that is a risk factor for aspiration: post swallow inhalation (Valenzano et al., 2020). Further, a previous study by our research group found changes in vocalization in the TgF344-AD model as early as 9 months (Rudisch et al., 2024), which suggests future work should look into the relationship between pathology in the nucleus ambiguus and vocalization changes.

The hypoglossal nucleus contains the motor neurons that innervate the tongue which initiates swallowing and manipulates the bolus during mastication—functions known to be impacted by Alzheimer’s (Mira et al., 2022; Seçil et al., 2016). Lingual weakness and reduced tongue function have been reported with clinical Alzheimer’s dysphagia (Simões et al., 2020; Ji et al., 2019). The tongue is often targeted by dysphagia interventions (Syahrul and Kadar, 2022; Rogus-Pulia et al., 2016), yet their effectiveness for Alzheimer’s dysphagia has not been established.

Brainstem pattern generators, circuits, and motor nuclei play a critical role in swallowing and mastication, yet only two brainstem regions of interest we evaluated were significant for Aβ. There are several different factors that may be important to consider regarding this lack of Aβ pathological findings in the brainstem.

The brainstem is one of the first regions to show hyperphosphorylated tau, yet Aβ depositions are not generally reported in the brainstem until Thal stage 4 (Thal et al., 2002), a later stage of Aβ deposition which corresponds to the point at which AD becomes symptomatic (Koychev et al., 2020). It is possible that our 11 month time point is at the cusp of Aβ deposition in the brainstem in the TgF344-AD model. While Aβ pathology has been identified as one potential factor in motor decline in AD (Andrade-Guerrero et al., 2024), tau pathology in the brainstem has been directly linked to neuronal loss and could be a driving force in functional changes. For example, the earliest tau pathology is found in brainstem nuclei, including the locus coeruleus (LC) and dorsal raphe nucleus (DRN) (Ehrenberg et al., 2017; Braak et al., 2011).

A detailed study by Parvizi et al. (2001) of post-mortem brainstem sections stained for both tau and Aβ revealed that AD-related changes occur only in certain nuclei and spare others. Thus, detecting meaningful differences in the brainstem may require spatially specific assays. A limitation of the current study is that the spatial resolution of the PET imaging (2 mm FWHM) is larger than several of our brainstem ROIs, particularly the locus coeruleus and the nucleus ambiguus (Figure 1; approximate dimensions LC: 1 mm × 0.5 mm × 0.2 mm, NA: 3.5 mm × 0.5 mm × 0.5 mm) (Paxinos and Watson, 2004; Pinos et al., 2001; Pascual-Font et al., 2011). Thus, their values are essentially being averaged with some of the surrounding tissue, potentially reducing the accuracy of the SUVr of these structures. While PET permits longitudinal within subject comparisons, it is possible that more direct tissue assays such as immunohistochemistry will be more sensitive to detecting functionally significant Aβ in these small structures, especially at earlier timepoints where the pathology has not reached peak severity.

Another limitation is that only male rats were used in this study. It is known that there are sex differences in specific brain regions and pathology progression in AD (Lopez-Lee et al., 2024). More specifically, differences between males and females have been reported in AB levels in this model, as detected by the same imaging approach as our study (Burgos-Puentes et al., 2026). Therefore, including both male and female groups would require substantially larger cohorts to have sufficient statistical power. In an effort to establish baseline PET swallowing biomarkers and constrain animal use in this initial study, we sought to examine our measurement endpoints in a male-only model with future expansion and comparison to female subjects.

## Conclusion

The TgF344-AD rat model of Alzheimer’s exhibits robust changes in oral processing and respiratory-swallow coordination that parallel clinical AD dysphagia. This study generated preliminary insights into brain regions impacted by Aβ pathology early in disease progression that may play a role in these swallowing changes. Future work should expand to include more time points and additional AD pathologies such as tau pathologies and neuronal loss.

Our finding of increased Aβ in the brainstem nucleus ambiguus warrants further study as this motor nucleus plays a role in swallowing, respiration, and vocalization—all factors that are known to be impacted by AD in the clinical population.

## Data Availability

The raw data supporting the conclusions of this article will be made available by the authors, without undue reservation.
