# Supplementary material for: Oropharyngeal dysphagia and amyloid beta pathology in the TgF344-AD rat model of Alzheimer’s disease
Source: Front Behav Neurosci. 2026 Apr 13;20:1812480. doi: 10.3389/fnbeh.2026.1812480 (PMC13111396; doi:10.3389/fnbeh.2026.1812480)
Supplement: Supplementary file 1 [file Table_1.DOCX]

| **Waxholm Atlas V1.01 region #** | **Waxholm Atlas V1.01 region name** | **p-value AD vs WT** |
| --- | --- | --- |
| 17 | Perirhinal cortex | 2.96E-07 |
| 86 | Hippocampal formation | 4.42E-06 |
| 87 | Subiculum | 3.36E-05 |
| 14 | Dorsal lateral entorhinal area | 4.62E-05 |
| 92 | Neocortex | 6.56E-05 |
| 67 | Corpus callosum and associated subcortical white matter | 9.61E-05 |
| 88 | Postrhinal cortex | 9.75E-05 |
| 6 | Alveus of the hippocampus | 0.0005 |
| 33 | Ventricular system | 0.0008 |
| 39 | Thalamus | 0.0010 |
| 91 | Caudal entorhinal field | 0.0011 |
| 50 | Superficial gray layer of the superior colliculus | 0.0019 |
| 31 | Globus pallidus | 0.0019 |
| 62 | Stria terminalis | 0.0023 |
| 59 | Fimbria of the hippocampus | 0.0049 |
| 94 | Pretectal region | 0.0052 |
| 55 | Deeper layers of the superior colliculus | 0.0062 |
| 68 | Brachium of the superior colliculus | 0.0112 |
| 1 | Descending corticofugal pathways | 0.0146 |
| 48 | Hypothalamic region | 0.0152 |
| 32 | Entopeduncular nucleus | 0.0153 |
| 34 | Medial lemniscus | 0.0165 |
| 15 | Dorsal intermediate entorhinal area | 0.0220 |
| 30 | Striatum | 0.0230 |
| 60 | Fasciculus retroflexus | 0.0236 |
| 52 | Fornix | 0.0248 |
| 83 | Supraoptic decussation | 0.0307 |
| 66 | Olfactory bulb | 0.0424 |
| 46 | Commissure of the superior colliculus | 0.0512 |
| 76 | Spinal trigeminal tract | 0.0552 |
| 78 | Middle cerebellar peduncle | 0.0568 |
| 82 | Basal forebrain region | 0.0616 |
| 49 | Inferior colliculus | 0.0652 |
| 42 | Optic tract and optic chiasm | 0.0680 |
| 74 | Inferior olive | 0.0724 |
| 38 | Ventral hippocampal commissure | 0.1050 |
| 51 | Periaqueductal gray | 0.1052 |
| 75 | Spinal trigeminal nucleus | 0.1308 |
| 58 | Pontine nuclei | 0.1356 |
| 37 | Anterior commissure posterior part | 0.1363 |
| 63 | Posterior commissure | 0.1419 |
| 53 | Mammillothalamic tract | 0.1486 |
| 61 | Stria medullaris of the thalamus | 0.1542 |
| 43 | Pineal gland | 0.1582 |
| 65 | Glomerular layer of the olfactory bulb | 0.1583 |
| 71 | Interpeduncular nucleus | 0.1646 |
| 45 | Spinal cord | 0.1764 |
| 79 | Transverse fibers of the pons | 0.1818 |
| 36 | Anterior commissure anterior part | 0.1950 |
| 80 | Habenular commissure | 0.1954 |
| 40 | Septal region | 0.1968 |
| 47 | Brainstem | 0.2234 |
| 90 | Medial entorhinal field | 0.2289 |
| 77 | Frontal association cortex | 0.2366 |
| 89 | Ventral intermediate entorhinal area | 0.2408 |
| 72 | Ascending fibers of the facial nerve | 0.2866 |
| 85 | Pyramidal decussation | 0.2996 |
| 35 | Facial nerve | 0.3033 |
| 73 | Anterior commissure intrabulbar part | 0.3250 |
| 81 | Nucleus of the stria medullaris | 0.3377 |
| 2 | Substantia nigra | 0.3400 |
| 3 | Subthalamic nucleus | 0.3435 |
| 54 | Commissural stria terminalis | 0.3436 |
| 5 | Deeper cerebellum | 0.3540 |
| 4 | Molecular cell layer of the cerebellum | 0.3540 |
| 44 | Inner ear | 0.3794 |
| 10 | Cingulate cortex area | 0.3948 |
| 56 | Periventricular gray | 0.4003 |
| 69 | Commissure of the inferior colliculus | 0.4089 |
| 64 | Glomerular layer of the accessory olfactory bulb | 0.4718 |
| 7 | Inferior cerebellar peduncle | 0.4759 |
| 41 | Optic nerve | 0.4778 |
| 93 | Bed nucleus of the stria terminalis | 0.4791 |
| 57 | Genu of the facial nerve | 0.4858 |

**Supplemental Table 1**: Our initial analysis focused on a priori analyses to limit false discoveries, but PET scans generated data on PIB uptake throughout the brain allowing for a more extensive exploratory analysis of the model. Using brain regions delineated by version 1.01 of the Waxholm space atlas, we generated a data set of AB levels (PIB uptake values), compared the WT and AD groups by student’s t-test (2-tailed), and ranked regions by p-value (right column).
