# Supplementary material for: Oropharyngeal dysphagia and amyloid beta pathology in the TgF344-AD rat model of Alzheimer’s disease
Source: Front Behav Neurosci. 2026 Apr 13;20:1812480. doi: 10.3389/fnbeh.2026.1812480 (PMC13111396; doi:10.3389/fnbeh.2026.1812480)
Supplement: Supplementary file 2 [file Table_2.DOCX]

| **Waxholm Atlas V4 region #** | **Waxholm Atlas V4 region name** | **p-value AD vs WT** |
| --- | --- | --- |
| 113 | Perirhinal area 36 | 5.3712E-07 |
| 98 | Cornu ammonis 1 | 8.48655E-07 |
| 112 | Perirhinal area 35 | 1.95161E-06 |
| 97 | Cornu ammonis 2 | 3.54359E-06 |
| 151 | Primary auditory area | 5.29401E-06 |
| 96 | Dentate gyrus | 7.61278E-06 |
| 100 | Subiculum | 8.16279E-06 |
| 153 | Secondary auditory area ventral part | 3.31914E-05 |
| 67 | Corpus callosum and associated subcortical white matter | 8.67212E-05 |
| 109 | Presubiculum | 9.88951E-05 |
| 108 | Postrhinal cortex | 0.00011 |
| 183 | Piriform cortex layer 3 | 0.00012 |
| 95 | Cornu ammonis 3 | 0.00012 |
| 406 | Secondary motor area | 0.00014 |
| 278 | Subparafascicular nucleus | 0.00021 |
| 152 | Secondary auditory area dorsal part | 0.00026 |
| 6 | Alveus of the hippocampus | 0.00033 |
| 266 | Ventral posterior nucleus of the thalamus parvicellular part | 0.00045 |
| 249 | External medullary lamina unspecified | 0.00048 |
| 268 | Retroreuniens thalamic nucleus | 0.00050 |
| 182 | Piriform cortex layer 2 | 0.00051 |
| 417 | Primary somatosensory area forelimb representation | 0.00061 |
| 287 | Zona incerta caudal part | 0.00062 |
| 33 | Ventricular system unspecified | 0.00066 |
| 443 | Secondary visual area lateral part | 0.00067 |
| 181 | Piriform cortex layer 1 | 0.00074 |
| 150 | Medial geniculate body marginal zone | 0.00078 |
| 281 | Subgeniculate nucleus | 0.00080 |
| 229 | Laterodorsal thalamic nucleus ventrolateral part | 0.00119 |
| 157 | External medullary lamina auditory radiation | 0.00120 |
| 422 | Secondary somatosensory area | 0.00122 |
| 282 | Limitans nucleus | 0.00152 |
| 204 | Pregeniculate nucleus | 0.00155 |
| 230 | Posterior thalamic nucleus | 0.00159 |
| 297 | Medial geniculate body medial division | 0.00174 |
| 50 | Superficial gray layer of the superior colliculus | 0.00186 |
| 444 | Temporal association cortex | 0.00190 |
| 501 | Amygdaloid area unspecified | 0.00197 |
| 68 | Brachium of the superior colliculus | 0.00206 |
| 420 | Primary somatosensory area face representation | 0.00209 |
| 442 | Primary visual area | 0.00215 |
| 198 | Globus pallidus external lateral part | 0.00216 |
| 283 | Lateral posterior thalamic nucleus lateral part | 0.00242 |
| 62 | Stria terminalis | 0.00304 |
| 210 | Posterior thalamic nuclear group triangular part | 0.00313 |
| 146 | Inferior colliculus brachium | 0.00329 |
| 228 | Laterodorsal thalamic nucleus dorsomedial part | 0.00383 |
| 227 | Ventral posteromedial thalamic nucleus | 0.00410 |
| 114 | Medial entorhinal cortex | 0.00425 |
| 433 | Parietal association cortex medial area | 0.00468 |
| 59 | Fimbria of the hippocampus | 0.00505 |
| 413 | Infralimbic area | 0.00548 |
| 411 | Cingulate area 1 | 0.00563 |
| 298 | Medial geniculate body ventral division | 0.00587 |
| 55 | Deeper layers of the superior colliculus | 0.00621 |
| 94 | Pretectal region | 0.00630 |
| 295 | Medial geniculate body dorsal division | 0.00658 |
| 286 | Lateral posterior thalamic nucleus medio caudal part | 0.00740 |
| 427 | Retrosplenial dysgranular area | 0.00748 |
| 299 | Medial geniculate body suprageniculate nucleus | 0.00760 |
| 138 | Lateral lemniscus intermediate nucleus | 0.00772 |
| 221 | Ventromedial thalamic nucleus | 0.00796 |
| 436 | Parietal association cortex posterior area | 0.00850 |
| 115 | Lateral entorhinal cortex | 0.00873 |
| 246 | Paracentral thalamic nucleus | 0.00930 |
| 222 | Submedius thalamic nucleus | 0.01058 |
| 34 | Medial lemniscus unspecified | 0.01082 |
| 430 | Retrosplenial granular area | 0.01101 |
| 235 | Zona incerta dorsal part | 0.01192 |
| 267 | Parafascicular thalamic nucleus | 0.01222 |
| 48 | Hypothalamic region unspecified | 0.01226 |
| 294 | Ventral posterolateral thalamic nucleus | 0.01236 |
| 416 | Granular insular cortex | 0.01309 |
| 408 | Primary motor area | 0.01336 |
| 432 | Parietal association cortex lateral area | 0.01360 |
| 425 | Primary somatosensory area barrel field | 0.01366 |
| 200 | Reticular prethalamic nucleus unspecified | 0.01377 |
| 500 | Endopiriform nucleus | 0.01482 |
| 1 | Corticofugal tract and corona radiata | 0.01501 |
| 195 | Globus pallidus external medial part | 0.01514 |
| 418 | Primary somatosensory area dysgranular zone | 0.01521 |
| 208 | Posterior intralaminar nucleus | 0.01633 |
| 239 | Pretectothalamic lamina | 0.01724 |
| 201 | Peripeduncular nucleus | 0.01805 |
| 290 | Intramedullary thalamic area | 0.01901 |
| 429 | Primary somatosensory area trunk representation | 0.01910 |
| 285 | Lateral posterior thalamic nucleus mediorostral part | 0.01950 |
| 32 | Entopeduncular nucleus | 0.01970 |
| 205 | Dorsal lateral geniculate nucleus | 0.02091 |
| 270 | Superior cerebellar peduncle and prerubral field | 0.02278 |
| 197 | Caudate putamen | 0.02304 |
| 52 | Fornix | 0.02429 |
| 164 | Reticular prethalamic nucleus auditory segment | 0.02499 |
| 60 | Fasciculus retroflexus | 0.02698 |
| 42 | Optic tract and optic chiasm | 0.02884 |
| 272 | Intergeniculate leaflet | 0.02997 |
| 216 | Rhomboid thalamic nucleus | 0.03069 |
| 236 | Zona incerta ventral part | 0.03077 |
| 424 | Agranular insular cortex posterior area | 0.03089 |
| 83 | Supraoptic decussation | 0.03162 |
| 110 | Parasubiculum | 0.03280 |
| 502 | Nucleus of the lateral olfactory tract | 0.03683 |
| 137 | Lateral lemniscus ventral nucleus | 0.03849 |
| 219 | Reuniens thalamic nucleus | 0.03860 |
| 448 | Secondary visual area medial part | 0.03892 |
| 423 | Primary somatosensory area hindlimb representation | 0.04172 |
| 238 | A13 dopamine cells | 0.04634 |
| 145 | Inferior colliculus external cortex | 0.04703 |
| 46 | Commissure of the superior colliculus | 0.05124 |
| 257 | Zona incerta rostral part | 0.05340 |
| 414 | Dysgranular insular cortex | 0.05451 |
| 76 | Spinal trigeminal tract | 0.05566 |
| 160 | Ventral cochlear nucleus cap area | 0.05567 |
| 159 | Ventral cochlear nucleus posterior part | 0.05612 |
| 199 | Ventral striatal region unspecified | 0.05697 |
| 78 | Middle cerebellar peduncle | 0.05723 |
| 232 | Mediodorsal thalamic nucleus lateral part | 0.05746 |
| 66 | Olfactory bulb unspecified | 0.05809 |
| 180 | Lateral olfactory tract | 0.05964 |
| 231 | Ventrolateral thalamic nucleus | 0.06339 |
| 223 | Angular thalamic nucleus | 0.06414 |
| 284 | A11 dopamine cells | 0.06621 |
| 248 | Central lateral thalamic nucleus | 0.06714 |
| 280 | Forel | 0.07196 |
| 74 | Inferior olive | 0.07236 |
| 213 | Anterodorsal thalamic nucleus | 0.07526 |
| 402 | Ventral orbital area | 0.08237 |
| 214 | Anteroventral thalamic nucleus dorsomedial part | 0.08532 |
| 51 | Periaqueductal gray | 0.08866 |
| 192 | Nucleus accumbens shell | 0.09019 |
| 38 | Ventral hippocampal commissure | 0.10498 |
| 35 | Facial nerve unspecified | 0.11059 |
| 122 | Vestibular nerve | 0.11121 |
| 247 | Central medial thalamic nucleus | 0.11900 |
| 82 | Basal forebrain region unspecified | 0.12391 |
| 405 | Prelimbic area | 0.12979 |
| 61 | Stria medullaris thalami | 0.13054 |
| 75 | Spinal trigeminal nucleus | 0.13083 |
| 233 | Mediodorsal thalamic nucleus central part | 0.13298 |
| 260 | Intermediodorsal thalamic nucleus | 0.13360 |
| 141 | Lateral lemniscus unspecified | 0.13425 |
| 58 | Pontine nuclei | 0.13556 |
| 218 | Xiphoid thalamic nucleus | 0.13570 |
| 53 | Mammillotegmental tract | 0.13645 |
| 143 | Inferior colliculus central nucleus | 0.13757 |
| 63 | Posterior commissure | 0.14186 |
| 37 | Anterior commissure posterior limb | 0.14407 |
| 184 | Nucleus accumbens core | 0.15392 |
| 43 | Pineal gland | 0.15818 |
| 65 | Glomerular layer of the olfactory bulb | 0.15831 |
| 71 | Interpeduncular nucleus | 0.16465 |
| 215 | Anteroventral thalamic nucleus ventrolateral part | 0.16853 |
| 123 | Ventral cochlear nucleus granule cell layer | 0.16938 |
| 163 | Nucleus sagulum | 0.17111 |
| 79 | Transverse fibers of the pons | 0.18155 |
| 410 | Agranular insular cortex dorsal area | 0.18221 |
| 409 | Agranular insular cortex ventral area | 0.18351 |
| 412 | Claustrum | 0.18943 |
| 130 | Trapezoid body | 0.19207 |
| 36 | Anterior commissure anterior limb | 0.19498 |
| 80 | Habenular commissure | 0.19540 |
| 40 | Septal region | 0.19670 |
| 131 | Nucleus of the trapezoid body | 0.20119 |
| 99 | Fasciola cinereum | 0.20254 |
| 47 | Brainstem unspecified | 0.20433 |
| 77 | Frontal association cortex | 0.20918 |
| 158 | Ventral cochlear nucleus anterior part | 0.21803 |
| 240 | Mediodorsal thalamic nucleus medial part | 0.22314 |
| 139 | Lateral lemniscus dorsal nucleus | 0.23055 |
| 242 | Paraventricular thalamic nuclei anterior and posterior | 0.24162 |
| 207 | Medial habenular nucleus | 0.24223 |
| 121 | Cochlear nerve | 0.24386 |
| 291 | Internal medullary lamina | 0.26198 |
| 206 | Lateral habenular nucleus | 0.27045 |
| 72 | Ascending fibers of the facial nerve | 0.28660 |
| 162 | Spiral ganglion | 0.29215 |
| 85 | Pyramidal decussation | 0.29960 |
| 3 | Subthalamic nucleus | 0.31357 |
| 407 | Frontal association area 3 | 0.31549 |
| 196 | Ventral tegmental area | 0.32294 |
| 120 | Cochlea | 0.32399 |
| 73 | Anterior commissure intrabulbar part | 0.32501 |
| 128 | Dorsal cochlear nucleus deep core | 0.32670 |
| 187 | Substantia nigra reticular part | 0.32694 |
| 119 | Vestibular apparatus | 0.32986 |
| 193 | Ventral pallidum | 0.33375 |
| 81 | Nucleus of the stria medullaris | 0.33767 |
| 54 | Commissural stria terminalis | 0.34356 |
| 404 | Dorsolateral orbital area | 0.34722 |
| 211 | Parataenial thalamic nucleus | 0.35390 |
| 4 | Molecular cell layer of the cerebellum | 0.35395 |
| 255 | Interanteromedial thalamic nucleus | 0.35837 |
| 142 | Inferior colliculus dorsal cortex | 0.36121 |
| 5 | Cerebellum unspecified | 0.36515 |
| 45 | Spinal cord | 0.38242 |
| 293 | Ventral anterior thalamic nucleus | 0.38886 |
| 140 | Lateral lemniscus commissure | 0.38910 |
| 126 | Dorsal cochlear nucleus molecular layer | 0.39231 |
| 10 | Cingulate area 2 | 0.39477 |
| 127 | Dorsal cochlear nucleus fusiform and granule layer | 0.39495 |
| 69 | Inferior colliculus commissure | 0.40502 |
| 188 | Substantia nigra compact part | 0.43801 |
| 125 | Th ventricle | 0.43816 |
| 56 | Periventricular gray | 0.44123 |
| 93 | Bed nucleus of the stria terminalis | 0.44558 |
| 132 | Superior paraolivary nucleus | 0.45004 |
| 401 | Lateral orbital area | 0.45194 |
| 254 | Anteromedial thalamic nucleus | 0.46080 |
| 400 | Ventrolateral orbital area | 0.46589 |
| 133 | Medial superior olive | 0.46808 |
| 189 | Substantia nigra lateral part | 0.46955 |
| 403 | Medial orbital area | 0.46992 |
| 64 | Glomerular layer of the accessory olfactory bulb | 0.47180 |
| 136 | Ventral periolivary nuclei | 0.47454 |
| 7 | Inferior cerebellar peduncle | 0.47492 |
| 41 | Optic nerve | 0.47776 |
| 57 | Genu of the facial nerve | 0.48576 |
| 135 | Superior periolivary region | 0.48796 |
| 129 | Acoustic striae | 0.48953 |
| 134 | Lateral superior olive | 0.49984 |

**Supplemental Table 2**: Our initial analysis focused on *a priori* analyses to limit false discoveries, but PET scans generated data on PIB uptake throughout the brain allowing for a more extensive exploratory analysis of the model. Using brain regions delineated by version 4 of the Waxholm space atlas, we generated a data set of AB levels (PIB uptake values), compared the WT and AD groups by student’s t-test (2-tailed), and ranked regions by p-value (right column).
